# Nicotine replacement therapy during the acute phase of aneurysmal subarachnoid hemorrhage

**DOI:** 10.1007/s00701-025-06698-y

**Published:** 2025-10-24

**Authors:** H. Ghafaji, A. Sorteberg

**Affiliations:** 1 University of Oslo, Institute of Clinical Medicine, Oslo, Norway; 2 Department of Neurosurgery, Oslo University Hospital, 4950, Nydalen, 0424 Oslo Norway

**Keywords:** Aneurysmal subarachnoid hemorrhage, Mortality, Nicotine replacement therapy, Outcome, Smoke, Vasospasm

## Abstract

**Background:**

Many patients who suffer an aneurysmal subarachnoid hemorrhage (aSAH) are active smokers that may experience nicotine withdrawal following hospital admission. Nicotine replacement therapy (NRT) could alleviate abstinence and delirium but may have unwanted side-effects. Cerebral vasospasm (VS) is a feared complication of aSAH that can worsen outcome. The impact of NRT on VS, complications and outcome is still not fully delineated.

**Methods:**

Retrospective study using anonymized data from a prospective quality registry. Patients smoking status, age, sex, comorbidities, along with aSAH severity were registered. Smokers were dichotomized into non-NRT and NRT groups depending on whether they had received a nicotine patch or not and subdivided into light smokers (≤ 10 cigarettes/day) and moderate to heavy smokers (> 10 cigarettes/day). We also registered radiological/sonological and clinical VS, delayed cerebral ischemia (DCI) related infarction and other common aSAH complications. Outcome was scored in terms of mortality and modified Rankin Score (mRS) at 90 days.

**Results:**

495 patients were included; 220 received NRT. NRT was not a predictor of radiological/ultrasonological VS or DCI-related infarction. Poor outcome was more frequent in light smokers when they had received NRT (12.49% vs 29.31%) and their length of hospitalization was longer. Moderate to heavy smokers that had received NRT developed less frequently atrial fibrillation (3.4% vs 11.7%) and their length of stay at the ICU was shorter. There was no difference in thromboembolic or epileptic events, or respiratory failure between groups. There was no difference in smoking cessation at 90 days with or without NRT.

**Conclusions:**

NRT had no impact on vasospasm or DCI-related infarction and it did not increase the frequency of complications. It seems advisable to abstain from NRT in light smokers.

**Supplementary Information:**

The online version contains supplementary material available at 10.1007/s00701-025-06698-y.

## Introduction

Despite advancements in management, aneurysmal subarachnoid hemorrhage (aSAH) still has a considerable mortality and morbidity [32, 46]. One of the most feared complications of aSAH is cerebral vasospasm (VS), a self-limiting narrowing of intracranial arteries. VS usually occurs multiple days after the aSAH and may cause delayed cerebral ischemia (DCI). VS can potentially worsen outcome and even lead to death [26, 27].

Smokers constitute a considerable amount of patients who suffer an aSAH [50]. Among our aSAH patients approximately 64% were smokers which is a much higher fraction than in the general Norwegian population in which 16% of males and 11% of females smoked in 2012, with a decline to 8% in both males and females in 2020 [15]. Despite smoking being so prevalent among patients with aSAH, there are no established recommendations or consensus handling nicotine dependence, and the potential abstinence that follows a hospital admission [8, 50]. Nicotine withdrawal has been associated with hyperactive, aggressive delirium [28, 34]. Moreover, nicotine withdrawal can highly mimic the symptoms of VS, thereby complicating clinical assessment and treatment [5]. This highlights the clinical challenge of distinguishing between true VS and nicotine withdrawal, which may have implications for treatment decisions. Withdrawal cannot only trigger or worsen delirium [50], but can also affect blood pressure levels, cardiac rhythm, and possibly the intracranial pressure negatively[39].

To reduce stressors like abstinence in aSAH patients, neurosurgeons and anaesthesiologists may administer NRT to smokers, whereas others may be reluctant to administer nicotine replacement therapy (NRT) due to the theoretical fear of inducing or worsening VS [8]. Still, the few studies linking smoking to VS [22, 54] have not explicitly mentioned nicotine in their proposed pathophysiological mechanisms. Instead, they suggest that the impact of smoking on cardiovascular VS might mimic that of VS. In comparison, a few studies noted that nicotine administration increased blood flow to multiple brain regions through a vasodilative effect [9, 35, 41, 55]. Thus, the effect of nicotine on cerebral blood vessels seems to contrast nicotine’s reported vasoconstrictive effects on extra-cerebral vessels [5].

The association between administration of NRT, the development of VS, and outcome remains controversial and understudied. Therefore, our study sought to contribute to fill the existing knowledge gap surrounding the relationship between NRT and the development of VS as well as the effect of NRT on outcome both in terms of mortality and functional outcome.

## Methods

### Patients

All patients admitted with aSAH to our neurosurgical tertiary centre between 2012 and 2020 were eligible. The following exclusion criteria were applied; non-smokers, age under 18 years, death within the first five days after the ictus (because of the time-frame in which VS develops), no aneurysm repair, unknown smoking history, and manifest VS at arrival to our department.

#### Research ethics

Research at Oslo University Hospital is conducted in accordance to the principles listed in the Declaration of Helsinki. For the present study data were acquired from an internal quality registry approved by the internal review board/data protection officer (approval # 11/6692). Data retrieval was approved by the internal review board/data protection officer as a quality project (approval # 21–10232) and we used anonymized data in our analysis. Consent was waived by the data protection officer in accordance with the Norwegian Patient Journal Act §6 and Health Personnel Act §26. The study did not receive any funding.

### Treatment principles and administration of NRT

Patients were treated according to our institutional algorithm [43]. Accordingly, with regard to VS, patients are screened with CTA and TCD on day 7 after the ictus, or day 5 if sedated and intubated. Thereafter, CT, TCD, or DSA was performed if there was clinical suspicion of VS. Upon admission, all patients received intravenous Nimodipine, which was changed to peroral administration of 60 mg × 6 as soon as patients could swallow. Patients were administered Nimodipine for a total of 21 days. If VS was diagnosed, the cerebral perfusion pressure (CPP) was increased from > 70 mmHg to > 90 mmHg. If neurological symptoms/malperfusion occurred despite of this, intraarterial (i.a.) spasmolysis was performed with Nimodipine.

Patients received NRT in the form of transdermal patches. Once a patient is identified as smoker, they may receive NRT early (usually within the first three days after admission) according to the preferences of the treating neurosurgeon or anesthesiologist. NRT is used irrespective of aSAH severity; i.e.in both awake and unconscious patients on respiratory support. NRT is not used as a means to treat agitation or delirium. None of the patients smoked during their hospital stay.

### Variables

The smoking status and amount was assessed at arrival. Information on amount of smoking was also gathered from next of kin during the primary admission as well as during follow-up. If there was inconsistency between amount, the quantity revealed during follow-up was entered into the database along with smoking status and amount at the time of follow-up. For the present study we subdivided the patients into light smokers (≤ 10 cigarettes/day) and moderate to heavy smokers (> 10 cigarettes/day) as per the definitions provided by the Canadian government [4]. Patients were further divided into those receiving a nicotine patch (NRT group) and those that did not receive NRT (Non-NRT group).

We registered the patients’ sex and age along with their clinical status after resuscitation and just prior to intubation using the World Federation of Neurosurgical Societies (WFNS) grading [37]. The WFNS score was then dichotomized into good grade (score 1–3) and poor grade (score 4–5). Based on the first available CT scan, we used the modified Fisher score [12] to measure the amount of SAH. The Fisher score was dichotomized into grade 1+ 2 versus grade 3 + 4. The amount of intraventricular hemorrhage (IVH) was expressed using the LeRoux score, 0 was assigned if there was no IVH [24]. Intraparenchymal hemorrhage was categorized according to maximum diameter (none, < 2 cm, 2–5 cm, > 5 cm). We also registered midline shift in mm and the localization of the index aneurysm. Coexisting medical disorders were noted and expressed using the Charlson Comorbidity Index [6]; a CCI score ≥ 2 was considered severe comorbidity [18]. We also registered the type of aneurysm repair and common complications in aSAH patients.

The maximum degree of VS diagnosed sonologically (Transcranial Doppler ultrasonography, TCD) or radiologically (computed tomography angiography, CTA) were scored as follows; no VS, slight to moderate VS in one vessel, slight to moderate VS in multiple vessels, severe VS in 1 vessel, and severe VS in multiple vessels. Clinical VS refers here to any delayed neurological worsening (i.e. new focal neurological deficit and/or drop in GCS beyond the fluctuation hitherto observed) which was not attributable to other complications [13]. If this is observed, institutional guidelines instruct us to exclude insufficiently treated hydrocephalus, rebleed, seizure, metabolic derangement inclusive electrolyte abnormalities, infection, fever, respiratory derangement, or medical sedative effects. DCI was defined radiologically as any ischemic lesion that was not due to the hemorrhage per se or a complication to aneurysm repair and is here denoted DCI-related infarction.

Mortality irrespective of cause was scored at 90 days. Functional outcome was scored around 90 days at the outpatient clinic using the modified Rankin scale, mRS [51]; a mRS of 0–2 was considered “good outcome” and mRS 3–6 “poor outcome”.

### Statistics

Statistical analyses were performed using SPSS v. 30.0.0.0 (IBM Corp., Armonk NY, USA). No imputation for missing data was performed. Normal distributed continuous variables are presented by mean and standard deviation, while continuous variables which were not normal distributed are presented with median and range. Categorical variables are presented in percentages. For group comparisons, categorical variables were analysed using Chi square test, normally distributed variables were analysed using T-tests and non-normally distributed variables were analysed by means of Mann–Whitney U test. We also performed a matched pairs analysis correcting for significant differences in entry variables between the NRT and non-NRT group. Uni- and multivariate analyses were carried out to identify predictors of poor outcome (mRS 3–6 at 90 days) and VS. Variables with p < 0.05 in the univariate analysis were included in the multivariate analysis. A two-sided p < 0.05 was considered significant.

## Results

### Patients

**Fig. 1:**
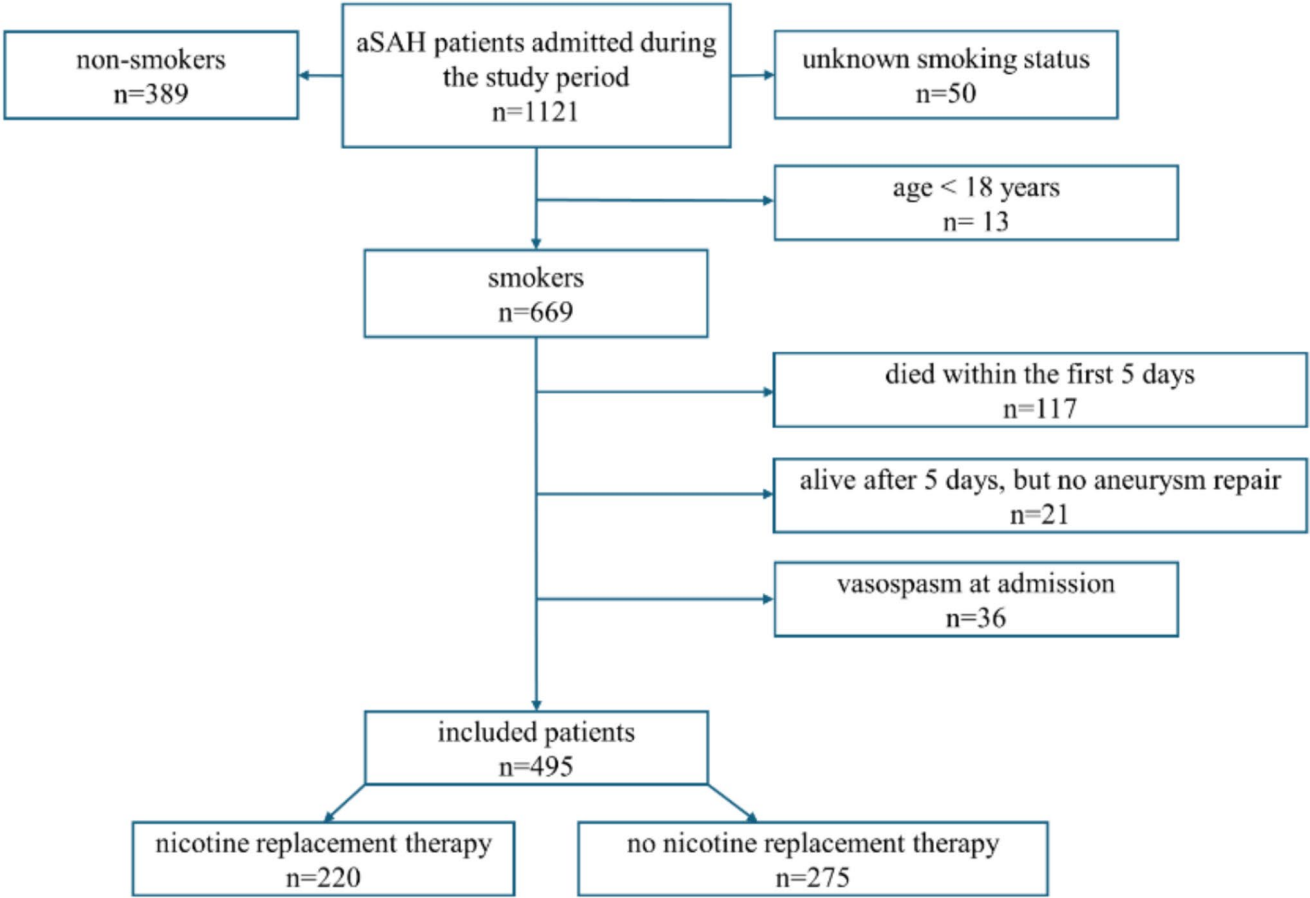
Flow chart of included patients

A total of 1115 patients were admitted with aSAH during the study period; of these, 495 patients were included in the study. Figure 1 shows a flow chart of included patients.

Among the included patients, 220 (44.4%) received NRT. Table 1 shows demographic, aSAH specific, and treatment characteristics for the NRT and non-NRT group. There were no significant differences between the groups except higher fraction of patients with severe comorbidity, higher amount of blood in all compartments, and hemicraniectomy being more frequent in the NRT group. MCA aneurysms were more common in the non-NRT group.

**Table 1:** Demographics, hemorrhage and treatment characteristics

Variable	Non-NRT Group (*N* = 275)	NRT Group (*N *= 220)	*p*-value
Light smokers	48.1%	22.1%	
Patient characteristics			
Age, years (mean ± SD)	56.3 ± 13.62	57.4 ± 11.22	0.401
Female (%)	64.4	58.2	0.160
**Comorbidity index (mean ± SD)** ** > 2 (%)**	0.80 ± 1.06 8.0	0.95 ± 1.22 13.6	0.234 **0.042**
Hypertension (%)	33.1	39.1	0.176
Clinical and radiological characteristics			
Rebleed prior to aneurysm repair (%)	6.9	3.6	0.111
**Acute subdural hematoma (%)**	**4.7**	**9.5**	**0.035**
Intraparenchymal hemorrhage (%)			
None	68.7	60.9	0.069
< 2 cm	10.5	15.0	0.137
2–5 cm	13.8	12.3	0.613
> 5 cm	6.9	11.8	0.059
WFNS 1–3	66.5	63.2	0.435
WFNS 4–5	33.5	36.8	0.435
Modified Fisher Scale (%)			
1 + 2	39.6	30.9	0.056
** 3 + 4**	60.4	69.1	**0.044**
Aneurysm localization (%)			
ACOM/A1/A2	37.4	43.2	0.196
ICA/PCOM	24.4	24.5	0.963
MCA	26.5	18.6	0.038
Vertebrobasilar	11.6	13.6	0.504
**LeRoux score (mean ± SD)**	**3.30 ± 3.973**	**3.92 ± 4.111**	**0.026**
Midline shift (mean ± SD)	1.29 ± 3.2	1.52 ± 3.33	0.503
Treatment			
Surgical aneurysm repair (%)	50.0	50.0	1
Permanent shunt treatment (%)	32.4	30.0	0.573
**Hemicraniectomy (%)**	**0.7**	**3.2**	**0.042**
Intraarterial spasmolysis (%)	8.4	11.8	0.201

*ACOM* anterior communicating artery, *ICA* internal carotid artery, *MCA* middle cerebral artery, *NRT* nicotine replacement therapy, *PCOM* posterior communicating artery, *WFNS* World Federation of Neurosurgical Societies

### Complications

Table 2 shows complications in the NRT and non-NRT group stratified by previous smoking amount and Table 3 shows the results for the same complications if patients were matched according to ASDH, modified Fisher Score, LeRoux Score and Charlson Comorbidity Index > 2. In light smokers, hospital and ICU admission times were longer in the NRT group, and females mainly accounted for this difference (supplemental Table 1). In contrast, all times were shorter in the NRT group among moderate to heavy smokers, this did, however, only reach significance for time in the ICU in the matched pairs analysis (Table 3).

**Table 2:** Hospital admission times and complications stratified by former smoking amount

Variable	Non-NRT group (*n* = 275)	NRT group (*n* = 220)	*p*-value
Light smokers (≤ 10 cigarettes/day)			
N =	116	43	
**Hospital stay (days) (Median, IQR)**	**12.8 (7.7, 17.2)**	**15.7 (12.8, 23.8)**	**0.002**
**Duration of intensive care unit stay (hours) (Median, IQR)**	**9.9 (7.0, 48.8)**	**26.8 (8.6, 203.8)**	**0.019**
**Length of respirator treatment (hours) (Median, IQR)**	**5.3 (3.7, 25.7)**	**13.0 (4.5, 119.3)**	**0.019**
Secondary respiratory failure (%)	5.2	4.7	0.894
De novo atrial fibrillation (%)	1.7	4.7	0.295
Thromboembolic event (%)	3.4	4.7	0.724
Epilepsy (%)	1.7	0.0	0.386
Clinical vasospasm (%)	17.2	14.0	0.619
Radiological/ultrasonological vasospasm (%)			
**No vasospasm**	**49.6**	**30.2**	**0.029**
Slight to moderate in 1 artery	10.4	18.6	0.169
Slight to moderate in multiple arteries	26.1	34.9	0.276
Severe in 1 artery	6.1	7.0	0.838
Severe multivessel spasm	7.8	9.3	0.764
DCI-related infarction (%)	8.6	11.6	0.564
**Poor outcome (modified Rankin Score 3–6)**	**12.4**	**29.3**	**0.013**
**90-day mortality (%)**	**1.8**	**10.0**	**0.021**
Smoking at follow-up (%)	52.9	46.2	0.556
Moderate to heavy smokers (> 10 cigarettes/day)			
N =	125	152	
Hospital stay (days) (Median, IQR)	16.9 (10.3, 21.8)	15.5 (11.7, 19.9)	0.882
Duration of intensive care unit stay (hours) (Median, IQR)	35.8 (7.9, 292.0)	26.3 (8.6, 180.0)	0.700
Length of respirator treatment (hours) (Median, IQR)	22.8 (4.5, 240.4)	17.1 (5.0, 170.7)	0.848
Secondary respiratory failure (%)	8.0	10.5	0.473
**De novo atrial fibrillation (%)**	**11.7**	**3.4**	**0.017**
Thromboembolic event (%)	7.2	8.6	0.679
Epilepsy (%)	0.8	0.7	0.889
Clinical vasospasm (%)	22.4	24.3	0.704
Radiological/ultrasonological vasospasm (%)			
No vasospasm	39.2	43.4	0.478
Slight to moderate in 1 artery	19.2	13.8	0.227
Slight to moderate in multiple arteries	21.6	25.0	0.506
Severe in 1 artery	8.0	5.9	0.496
Severe multivessel spasm	12.0	11.8	0.968
DCI-related infarction (%)	16.8	13.8	0.491
Poor outcome (modified Rankin Score 3–6)	23.5	24.1	0.912
90-day mortality (%)	8.1	9.6	0.661
Smoking at follow-up (%)	62.5	60.9	0.831

*DCI* delayed cerebral ischemia, *NRT* nicotine replacement therapy, *VS *vasospasm. Significant differences in bold

**Table 3:** Pairs matched for acute subdural hematoma, Charlson comorbidity index > 2, modified Fisher Score and amount of intraventricular blood (Le Roux Score)

Variable	Non-NRT group	NRT group	*p*-value
Light smokers (≤ 10 cigarettes/day), N = 43 pairs			
**Hospital stay (days) (Median, IQR)**	**13.1 (9.8;17.2)**	**15.7 (12.8;23.9)**	**0.022**
Duration of intensive care unit stay (hours) (Median, IQR)	16.4 (7.8;72.7)	26.8 (8.6;211.6)	0.349
Length of respirator treatment (hours) (Median, IQR)	5.5 (3.7;44.1)	13.0(4.4;120.0)	0.154
Secondary respiratory failure (%)	9.3	4.7	0.397
De novo atrial fibrillation (%)	4.7	4.7	1.000
Thromboembolic event (%)	2.3	4.7	0.557
Epilepsy (%)	2.3	0	0.314
Clinical vasospasm (%)	16.3	14.0	0.763
Radiological/ultrasonological vasospasm (%)			
No vasospasm	42.9	30.2	0.227
Slight to moderate in 1 artery	16.7	18.6	0.815
Slight to moderate in multiple arteries	31.0	34.9	0.700
Severe in 1 artery	4.8	7.0	0.664
Severe multivessel spasm	4.8	9.3	0.414
DCI-related infarction (%)	2.3	11.6	0.090
Poor outcome (modified Ranking Score 3–6)	18.6	27.5	0.335
90-day mortality (%)	2.3	9.3	0.167
Smoking at follow-up (%)	27.9	27.8	1.000
Moderate to heavy smokers (> 10 cigarettes/day), N = 99 pairs			
Hospital stay (days) (Median, IQR)	17.1(10.5;22.1)	15.2(10.6;19.8)	0.143
**Duration of intensive care unit stay (hours) (Median, IQR)**	**49.5(8.6;319.2)**	**26.3(8.6;180.0)**	**0.047**
Length of respirator treatment (hours) (Median, IQR)	27.7(5.0;284.8)	16.2(5.2;185.4)	0.166
Secondary respiratory failure (%)	9.1	10.1	0.809
**De novo atrial fibrillation (%)**	**13.1**	**2.0**	**0.003**
Thromboembolic event (%)	8.1	7.1	0.788
Epilepsy (%)	1.0	1.0	1.000
Clinical vasospasm (%)	26.3	25.3	0.871
Radiological/ultrasonological vasospasm (%)			
No vasospasm	36.4	44.4	0.247
Slight to moderate in 1 artery	19.2	11.1	0.113
Slight to moderate in multiple arteries	23.2	27.3	0.513
Severe in 1 artery	7.1	3.0	0.194
Severe multivessel spasm	14.1	14.1	1.000
DCI-related infarction (%)	19.2	15.2	0.451
90-day mortality (%)	8.6	12.2	0.422
Poor outcome (modified Ranking Score 3–6)	23.9	25.6	0.797
Smoking at follow-up (%)	36.8	35.8	0.866

*DCI* delayed cerebral ischemia, *NRT* nicotine replacement therapy, *VS* vasospasm. Significant differences in bold

De novo atrial fibrillation occurred significantly less in the NRT group among moderate to heavy smokers (3.4% versus 11.6%, *p* = 0.017), and this finding was even more pronounced in the matched pairs analysis (Table 3). There were no statistically significant differences in the occurrence of secondary respiratory failure, thromboembolic events or epilepsy between non-NRT and NRT group, irrespective of smoking amount.

#### Vasospasm

Complete absence of radiological/ultrasonological VS was seen more often in the whole non-NRT group among light smokers, but this could not be reproduced in the matched pairs analysis (Tables 2 and 3). There were no statistically significant differences in clinical VS or DCI-related infarction. Among moderate to heavy smokers NRT was not associated with radiological/ultrasonological VS, clinical VS, or DCI-related infarction.

For all 495 patients, NRT was not a predictor of clinical VS (*p* = 0.473), radiological/ultrasonological VS (*p* = 0.418) or DCI (*p* = 0.626). These results remained unchanged when stratifying by smoking amount.

### Outcome

**Fig. 2:**
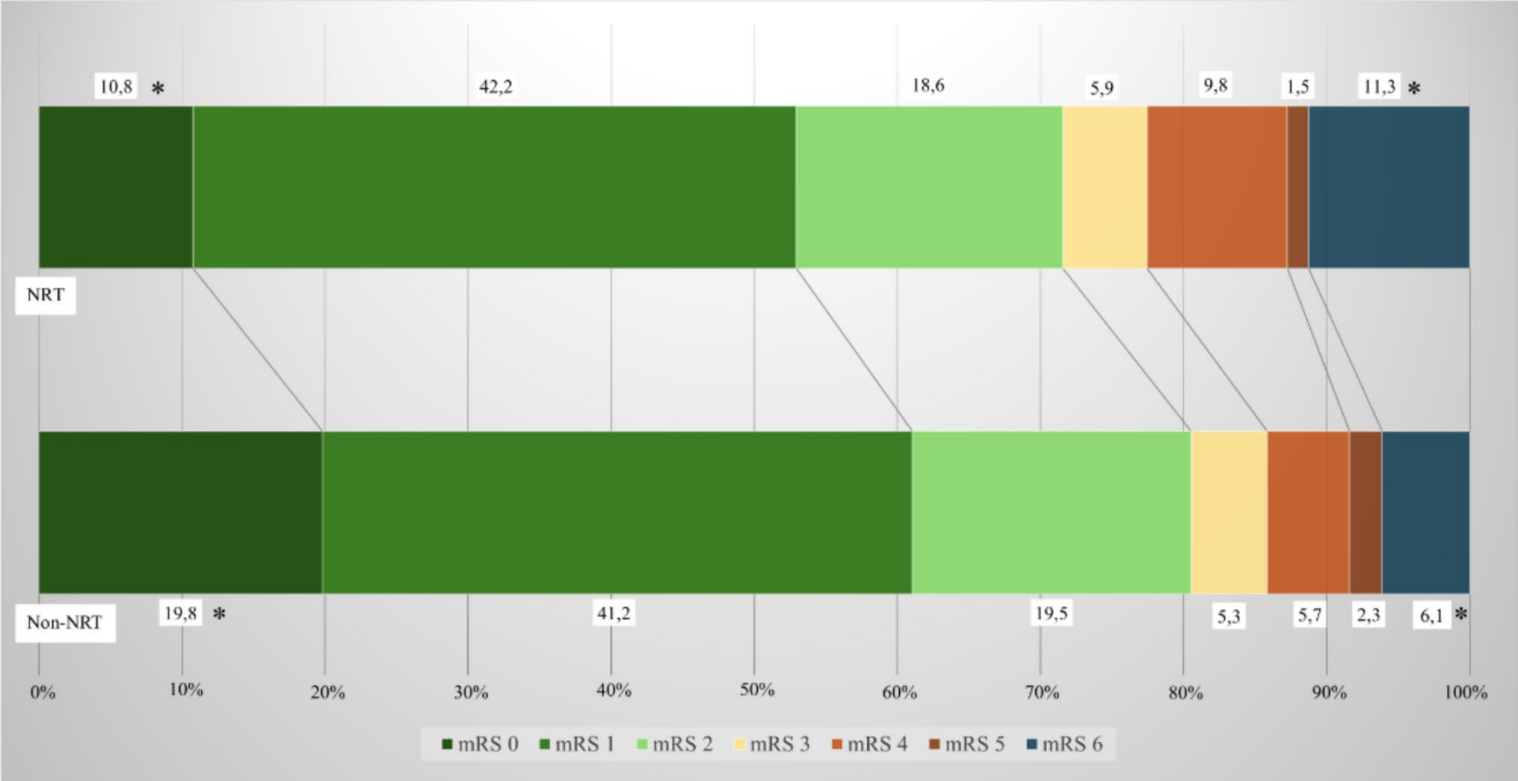
Stacked bar chart of modified ranking score in those that received nicotine replacement therapy (upper) and those that did not (lower) for the entire study cohort *: *p* < 0.05

For all 495 patients, excellent outcome with mRS 0 was more prevalent in the non-NRT group (*p* = 0.008). Likewise, good outcome (mRS 0–2) was seen more often in the non-NRT group (80.5% vs 71.6%, *p* = 0.023). Figure 2 shows outcome in both groups.

**Fig. 3:**
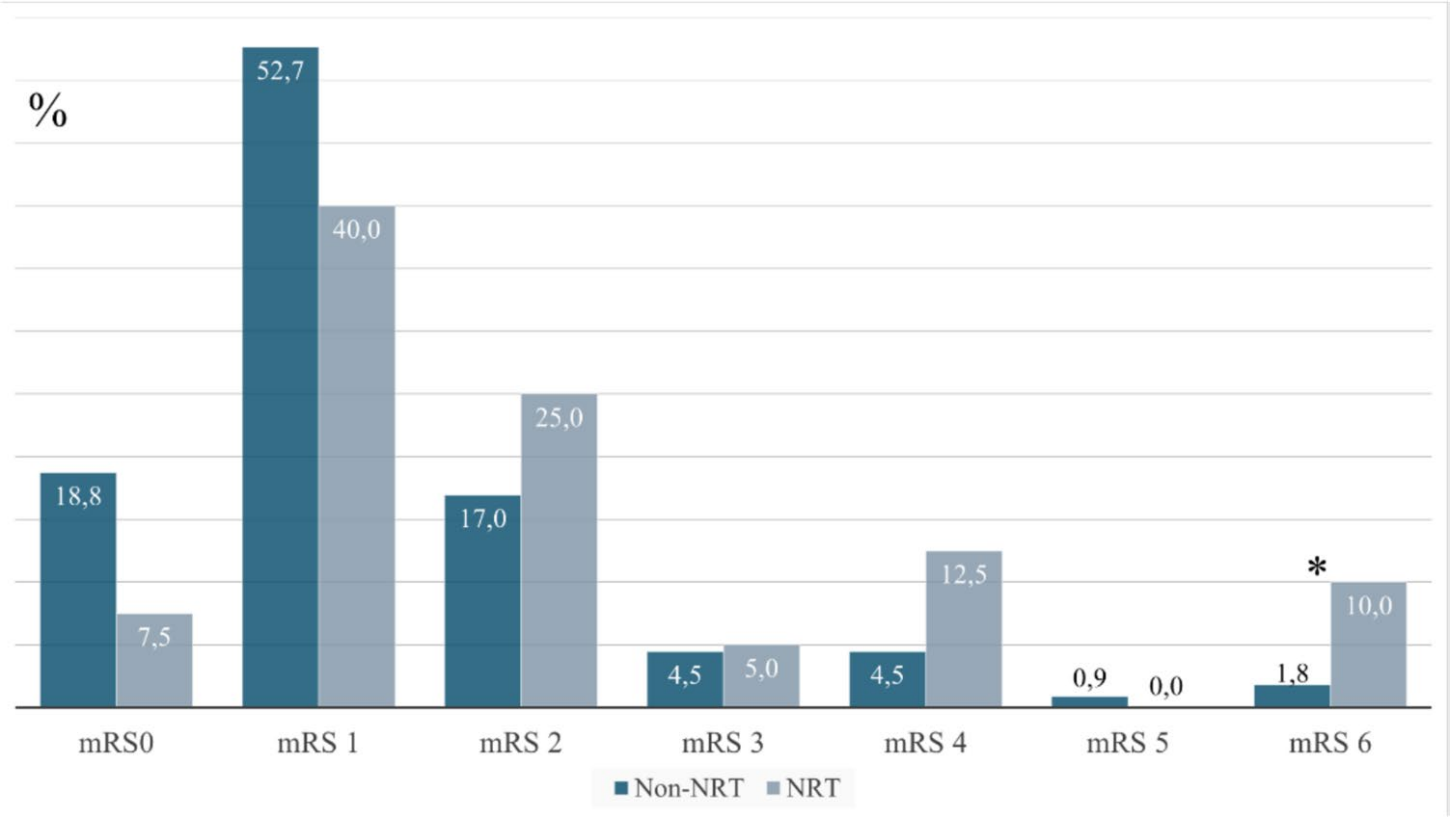
Modified rankin score in light smokers with NRT (light blue columns) and without NRT (dark blue columns), *: *p* < 0.05

Among light smokers, poor outcome was higher in the NRT group (Tables 2 and 3, Fig. 3). This finding was attributable to the subgroup of male light smokers with NRT and was not observed in females (Supplemental Table 1). There was no statistically significant difference in poor outcome between the non-NRT and NRT group in moderate to heavy smokers (Tables 2 and 3). 90-day mortality was higher in the NRT group among light smokers (particularly in males), but not among moderate to heavy smokers.

There was no statistically significant difference in smoking cessation between non-NRT and NRT among light smokers or in moderate to heavy smokers, Tables 2 and 3.

Table 4 shows the uni- and multivariate analysis for predictors of poor outcome for the entire study cohort. Higher age, WFNS, comorbidity, IVH, ICH, DCI-related infarction, and secondary respiratory failure were independent predictors of poor outcome, whereas NRT was not.

**Table 4:** Uni- and multivariate analysis of predictors of poor outcome (modified Ranking Score 3–6 at 90 days)

	Univariate analysis		Multivariate analysis	
	Odds ratio (95% CI)	*p*-value	Odds ratio (95% CI)	*p*-value
**NRT**	**1.644 (1.068, 2.530)**	**0.024**		
**Age, years**	**1.065 (1.044, 1.087)**	** < 0.001**	**1.085 (1.049,1.122)**	** < 0.001**
Female	1.270 (0.810, 1.992)	0.297		
**Comorbidity index**	**1.545(1.286,1.855)**	** < 0.001**	**1.354 (1.008, 1.819)**	**0.044**
**Hypertension**	**2.245 (1.439,3.500)**	** < 0.001**		
**Rebleed prior to aneurysm repair**	**3.557 (1.549, 8.167)**	**0.003**		
**ICH**	**2.126 (1.735, 2.605)**	** < 0.001**	**1.367 (1.008, 1.855)**	**0.045**
**WFNS**	**2.442 (2.032, 2.936)**	** < 0.001**	**2.198 (1.676, 2.881)**	** < 0.001**
**Modified Fisher 3 + 4**	**3.354 (1.959, 5.743)**	** < 0.001**		
Aneurysm location ACom/A1/A2 MCA ICA/PCom Vertebrobasilar	0.885 (0.570,1.374) 0.932 (0.557, 1.561) 0.910 (0.548, 1.511) 1.660 (0.905, 3.048)	0.587 0.789 0.715 0.102		
**Le Roux (IVH)**	**1.265 (1.196, 1.339)**	** < 0.001**	**1.119 (1.032, 1.214)**	**0.007**
Surgical aneurysm repair	0.971(0.632, 1.491)	0.893		
Permanent shunt treatment	1.355 (0.861, 2.133)	0.189		
**Secondary respiratory failure**	**6.786 (3.427, 13.437)**	** < 0.001**	**3.681 (1.402, 9.665)**	**0.008**
**De novo atrial fibrillation**	**3.557 (1.549, 8.167)**	**0.003**		
**Thromboembolic events**	**3.401 (1.622, 7.132)**	**0.001**		
Epilepsy	1.992 (0.468, 8.475)	0.351		
Clinical VS	0.778 (0.451, 1.342)	0.367		
VS (CTA/TCD)	1.038 (0.888, 1.215)	0.638		
Severe VS in one or multiple vessels	1.029 (0.590,1.793)	0.920		
**DCI-related infarction**	**3.170 (1.823, 5.514)**	** < 0.001**	**4.191 (1.676, 10.479)**	**0.002**

All patients, *n* = 495

Variables included in the multivariate analysis are emphasized in bold. Only variables that remained significant in the multivariate analysis are shown. *ACOM* anterior communicating artery, *DCI* delayed cerebral ischemia, *ICA* internal carotid artery, *MCA* middle cerebral artery, *PCOM* posterior communicating artery, *VS* vasospasm, *WFNS* World Federation of Neurosurgical Societies

In light smokers, NRT was a significant predictor of poor outcome in the univariate analysis (OR 2.926, 95% CI 1.220–7.020, *p* = 0.016) and borderline significant in the multivariate analysis (OR 3.245, 95% CI 0.911, 10.621, *p* = 0.052). Significant independent predictors of poor outcome in that subgroup were age (OR 1.081, 95% CI 1.029, 1.135, *p* = 0.002), ICH (OR 1.996, 95% CI 1.253, 3.180, *p* = 0.004) and LeRoux score (OR 1.402, 95% CI 1.186, 1.658, *p* < 0.001). In moderate to heavy smokers, multivariate analysis identified age (OR 1.107, 95% CI 1.063, 1.153, *p* < 0.001), DCI-related infarction (OR 3.694, 95% CI 1.228, 11.108, *p* = 0.020), WFNS (OR 2.480, 95% CI 1.776, 3.463, *p* < 0.001), secondary respiratory failure (OR 3.325, 95% CI 1.022, 10.821, *p* = 0.046) and LeRoux score (OR 1.147, 95% CI 1.046, 1.257, p = 0.004) as independent predictors of poor outcome. NRT was not a predictor of outcome in the univariate analysis (*p* = 0.816) and was therefore not entered in the multivariant analysis.

## Discussion

The core finding of this study was that NRT has no impact on VS. NRT was associated with higher 90-day mortality, poorer outcomes and prolonged hospitalization in light smokers. Moderate to heavy smokers receiving NRT exhibited significantly lower rates of de novo atrial fibrillation, whereas there was no impact of NRT on other complications or outcome. NRT had no impact on smoking cessation at follow-up, regardless of smoking amount.

### NRT and vasospasm

The definition and diagnosis of VS varies in the literature and one can assume differences between studies that are caused by different methods to establish the presence of VS. We therefore scored our patients both for radiological/ultrasonographical VS, clinical VS (which many denote as DCI) and radiologically ischemic lesions (here defined as DCI-related infarction). NRT was not associated with VS among moderate to heavy smokers, regardless of how it was measured. In light smokers, radiological/ultrasonological VS was more often absent without NRT. However, it is unlikely that a finding of no VS versus light or moderate VS has any clinical implication or effect on outcome. Our findings concur with those from other reports that did not find an association between NRT and increased frequency or severity of angiographic VS [5, 33, 39], or clinical VS [5]. In these studies, VS was either diagnosed using digital subtraction angiography (DSA) [5] or TCD [5, 39], though Panos et al. did not specify which modality was applied for assessing VS [33]. Carandang et al. even reported less clinical VS with NRT, where clinical VS was defined as any radiological narrowing of a vessel (either > 25% from baseline or > 50% from normal) with an associated clinical deterioration not attributable to any other cerebral or non-cerebral complications with the latter corresponding to what we presently denoted as clinical VS [5]. Moreover, Seder et al. is the only study to mention DCI in relation to NRT, and they did not find DCI more often with NRT [39]. DCI was in that study defined as ‘clinical deterioration or cerebral infarction due to vasospasm’[39].

There are reports of nicotine exerting both vasodilatory and vasoconstrictive effects. Administration of nicotine has been associated with increased cerebral blood flow in chronic smokers [9, 35, 41]. Given that vasoconstriction and endothelial dysfunction contribute to the development of VS [1, 8, 19], it seems reasonable to assume that nicotine’s vasodilatory effects could counteract these mechanisms. Nicotine may hence reduce the severity of hypoperfusion, potentially lowering the risk and extent of DCI [7, 19]. On the other hand, chronic smoking has also been associated with reduced cerebral blood flow, primarily due to nicotine’s inhibitory effect on nitric oxide (NO) synthesis, a mechanism implicated in the pathogenesis of VS [9, 21, 47, 49]. However, most studies have not stratified their findings by smoking amount, making it difficult to determine whether the observed vasoactive effects were influenced by cumulative nicotine exposure. This lack of differentiation complicates the interpretation of results and highlights the need for a more nuanced understanding of nicotine’s impact on the cerebral circulation.

One may argue that nicotine’s vasoactive properties are highly dependent on prior nicotine exposure, which could explain the different trends observed in light smokers compared to moderate to heavy smokers. Elbejjani et al. found that higher pack-years were associated with significantly increased cerebral blood flow among current smokers [9]. This may be due to smokers developing chronic obstructive lung disease which lowers their blood oxygen levels and may trigger a compensatory rise in cerebral blood flow in order to maintain an adequate brain tissue oxygenation. Another explanation for increased cerebral blood flow in smokers is that prolonged exposure to nicotine may induce vascular adaptations that shift nicotine’s effects toward vasodilation when administered in low doses [9, 35, 41]. However, what constitutes a “low dose” is relative to prior nicotine exposure—what may be considered a low dose for moderate to heavy smokers could be excessive for light smokers. This could explain why NRT had a slight association with VS in light smokers, as their lower baseline nicotine levels may have made them more susceptible to the unwanted effects of nicotine. Conversely, in moderate to heavy smokers, the same NRT doses may have instead promoted nicotine’s vasodilatory effects, mitigating VS risk [9]. Still, if nicotine exerted a pronounced vasodilatory effect, one would expect a significantly lower incidence of VS among moderate to heavy smokers receiving NRT. Since this was not the case, it could be argued that the administered dose was sufficient to partially mitigate VS formation rather than completely counteract it. This raises further questions about the threshold at which nicotine transitions from a vasoconstrictive to a vasodilatory agent, which likely depends on both prior exposure levels and the acute dosing regimen.

It seems worthwhile to point out that nicotine is not synonymous with tobacco smoke. While nicotine is one of the key vasoactive components in tobacco, it is only one of many substances that may contribute to the development of VS and DCI. Some studies challenge the idea that nicotine alone is integral to the formation of VS [1, 5, 8], suggesting that other toxic components in tobacco smoke may play a more significant role [5, 50]. These include carbon monoxide and polycyclic aromatic hydrocarbons [10], among more than 7000 other chemicals present in tobacco smoke [42]. Both carbon monoxide and polycyclic aromatic hydrocarbons are associated with increased oxidative stress and inflammation [17, 31, 36], which in turn may alter cerebrovascular tone and promote endothelial dysfunction [17, 31]. One should acknowledge the impact of the noxious components found in tobacco smoke when discussing the relationship between NRT and VS, because nicotine when administered as NRT is not equal to nicotine interacting with possibly 7000 other chemicals in tobacco smoke. While nicotine itself can exert vasoactive effects that depend on prior nicotine exposure [9, 21, 47, 49], its role in the development of VS appears to be modulated by the presence of these additional toxic compounds. It is plausible that nicotine acts synergistically with these substances in tobacco smoke to drive endothelial dysfunction, sympathetic activation, and vasoconstriction, ultimately leading to VS in smokers. This potential synergy could in part explain why NRT was not associated with increased VS in moderate to heavy smokers, as nicotine alone may not exert the same degree of vasoconstrictive influence as it does when combined with the other constituents of tobacco smoke.

### NRT and complications other than VS

Presently, NRT was associated with less development of atrial fibrillation in moderate to heavy smokers. aSAH can trigger both arrhythmias, including atrial fibrillation, and ECG disturbances, with an estimated 50–100% of patients experiencing the latter in the acute phase [14, 16]. Nicotine withdrawal is associated with dysregulation of the hypothalamic–pituitary–adrenal axis end thereby with the stress-response system [20]. This stress may increase the vulnerability for atrial fibrillation. This effect would be strongest in those used to the highest nicotine blood levels. NRT did not seem to increase the risk of de novo atrial fibrillation in aSAH in light smokers. A few reports proposed a link between atrial fibrillation and NRT, but they were conducted on patients who smoked concurrently with NRT use or who used NRT improperly [29, 38, 45]. None of our patients smoked during their hospital admission.

NRT did not lead to an increased occurrence of respiratory failure and thromboembolism. This phenomenon was observed irrespective of smoking amount and aligns with prior studies on side effects of NRT [29]. During the first day of smoking cessation, carbon monoxide in the blood decreases to normal levels which leads to swelling of erythrocytes in smokers and thereby enhances the risk of thromboembolism during that time frame. It is uncertain if this is due to nicotine or rather other compounds in smoke. Our results do not support any notion of reduced thromboembolic risk with nicotine substitution. A study on NRT and post-operative complications found no overall increase in post-operative complications, including respiratory failure [44]. Approximately 24 h after smoking cessation, the respiratory cilia regain better function and there is a higher production and transportation capacity of mucus which would cause increased coughing in awake smokers. In patients with reduced consciousness this can lead to secretory stagnation and increased respiratory problems. If nicotine plays a significant role in this effect, its replacement could theoretically reduce time on mechanical respiratory support in poor grade patients. We actually observed shorter times for ICU stays and respiratory support in our moderate to heavy smokers that received NRT. It is, however, questionable whether this is attributable to a nicotine effect in the lungs or due to lower stress levels with NRT, allowing extubation at an earlier point of time.

### NRT and outcome

NRT was associated with poor outcome in our light smokers. Since NRT was not associated with DCI-related infarction or VS, it seems likely that NRT affected outcome through other mechanisms than those related to DCI. While no clinical studies have directly addressed this, animal and experimental models suggest that chronic nicotine exposure may alter the cerebral microcirculation and induce microscopic thromboemboli [2, 11, 23, 53]. This microvascular dysfunction would, however, also occur in moderate to heavy smokers in whom we did not see a negative effect from NRT on outcome. If NRT is able to exacerbate microcirculatory dysfunction independent of VS, the lack of this effect in moderate to heavy smokers raises questions. A plausible explanation is that the impact of nicotine on cerebral vessels is influenced by prior nicotine exposure, as chronic exposure could alter nicotinic receptor expression and downstream receptor-mediated effects in ways that modify vascular response.

If nicotine has vasodilatory effects on intracerebral vessels, it will also lead to an increase in cerebral blood volume and thereby ICP. This effect would be most prominent in the light smokers that are administered relatively high doses of nicotine. Increased ICP would be managed according to protocol so that one can assume that the ICP did not reach levels that would impact outcome negatively. It could, however, prolong hospitalization times as we saw in our light smokers.

Inflammatory responses following stroke, including aSAH, are also relevant when considering outcome differences based on smoking amount. The literature on nicotine’s role in post-stroke inflammation is conflicting. Some studies conducted on animal models have found that low dose nicotine exerts a protective effect on neuronal tissue by attenuating cytotoxicity, minimizing tissue injury and reducing cell death in the case of cerebral ischemia [30, 52]. Nicotine has also been implicated in neuroplasticity and induction of neurotrophic factors, potentially improving cognitive function [3, 48]. On the other hand, there are also studies that associate nicotine with exacerbated inflammatory responses following stroke, including brain edema and reperfusion injuries [2, 25, 40]. Both chronic and acute exposure of nicotine were associated with pro-inflammatory mechanisms [2, 25, 40]. Notably, the levels of nicotine exposure in these studies vary widely, complicating the interpretation of dose-dependent effects. This variability underscores the importance of considering prior nicotine exposure when assessing the vascular and inflammatory consequences of NRT, as pre-existing receptor adaptations and systemic responses may modulate its effects.

Exposure to nicotine through cigarette smoke is different from the continuous supply provided by a nicotine patch. When smoking, there would be a nighttime break of nicotine exposure which is not the case with a nicotine patch. Smoking 5 cigarettes/day leads to approximately 1.5 mg × 5 of nicotine entering the body, which is far less than the daily dose of a patch of 14–21 mg. Light smokers may hence be exposed to higher doses of nicotine than they are used to which could lead to higher blood pressure and heartrate, nausea and vomiting, headache, as well as hyperventilation. In contrast to that, in moderate to heavy smokers, the nicotine patch is a more adequate substitution of the nicotine deficit arising with smoking cessation upon hospitalization.

If assuming a positive effect of NRT, one may also argue for the opposite, i.e. not a situation of too much nicotine but too little nicotine contributing to poorer outcome. Nicotine lowers stress and pain by reducing dopamine in the brain, an action that seems to be dependent on the endogenous opioid system [20]. Substances that counteract the opioid system, such as Naloxone, would hinder nicotine’s stress reduction. In aSAH patients that are able to swallow, we often use painkillers with the combination of oxycodone/naloxone, which could then either reduce or nullify nicotine’s protective effects against stress and withdrawal symptoms, thereby potentially worsening delirium.

Carandang et al. [5] have not seen a negative impact on outcome with NRT, however, their NRT group had less severe aSAH, whereas our NRT patients had a higher severity. Moreover, they scored outcome at discharge, whereas we scored it at 90 days and we expressed outcome with the mRS which is more nuanced than the Glasgow Outcome Score that Carandang et al. used [5]. Panos et al. found that NRT was not associated with increased VS when controlling for aSAH [33], and Seder et al. reported that NRT was associated with lower mortality [39]. However, neither stratified their patients by smoking amount, complicating the interpretation of NRT on VS and mortality.

NRT has been considered a useful tool in permanent smoking cessation. Still, in the setting of NRT in acute aSAH, there was no difference between the non-NRT and NRT group regarding smoking cessation. This may be attributable to the patients being forced into smoking cessation and not having chosen to quit themselves. While some patients report to have lost the craving for cigarettes after their aSAH, others fall back into their usual habits once they return to their home and may even smoke more due to inability to work and boredom.

### Strengths and limitations

The retrospective character of the study is a clear limitation; however, data are from a prospective database assumed to contain relatively high quality data. Our study includes more than twice the number of those included in earlier studies (that also were retrospective). The single-centre design of the study limits its external validity; especially outcome is a complex multifactorial construct that would be affected by institutional treatment protocols and possibly modify the effect of NRT. A strength is that we also took into consideration the intensity of chronic nicotine exposure prior to aSAH, which unveiled somewhat different risks in light versus moderate to heavy smokers. Still, the amount of cigarettes smoked may have been underreported by patients. Furthermore, precise data on the duration of nicotine exposure prior to ictus was not available, which is unfortunate as it could have provided valuable insights into potential dose-dependent effects of nicotine on VS, DCI-related infarction, and functional outcomes. A strength is that none of the included patients actively smoked during hospitalization, minimizing the confounding effects of tobacco smoke. This is particularly important, as previous studies examining the relationship between nicotine/NRT, VS and extra-cerebral complications have not always distinguished between the effects of nicotine itself and those of tobacco smoke.

We did not systematically assess nicotine withdrawal symptoms, preventing us from evaluating the efficacy of NRT in mitigating withdrawal-related complications. We also had no measurements of delirium or important medical variables like heart rate, blood pressure, and ICP levels available within this study. Even with evaluation of delirium it would be difficult to univocally state its cause; i.e. delineate delirium as an adverse event of NRT and distinguish it against the delirium we frequently see in aSAH patients. Finally, NRT dosing and selection for NRT was not standardized and was decided by the treating physician, introducing the possibility of treatment bias. Future studies should preferably be carried out in a randomized controlled design.

## Conclusion

NRT was not an independent predictor of VS or DCI-related infarction, irrespective of smoking amount. Occurrence of de novo atrial fibrillation was significantly less in moderate to heavy smokers with NRT, whereas occurrence of respiratory failure and thromboembolic events were similar between the non-NRT and NRT group. Poor outcome was more common in light smokers that received NRT and their hospital stay was prolonged, so that we would advise to abstain from NRT.

## Supplementary Information

Below is the link to the electronic supplementary material.

Supplementary file1 (DOCX 17 KB)

## Data Availability

Data from the present study can be made available upon reasonable request.
