# Supplementary material for: Nicotine replacement therapy during the acute phase of aneurysmal subarachnoid hemorrhage
Source: Acta Neurochir (Wien). 2025 Oct 24;167(1):281. doi: 10.1007/s00701-025-06698-y (PMC12552259; doi:10.1007/s00701-025-06698-y)
Supplement: Supplementary file 1 — Supplementary file1 (DOCX 17 KB) [file 701_2025_6698_MOESM1_ESM.docx]

Supplemental Table 1: *Hospital admission and complications stratified by former smoking amount and gender for smokers in the Non-NRT group and the NRT group.*

| Variable | Non-NRT group  female | NRT group  female | p-value | Non-NRT group  male | NRT group  male | p-value |
| --- | --- | --- | --- | --- | --- | --- |
| Light smokers (≤10 cigarettes/day) | | | | | | |
| N= | 87 | 31 |  | 29 | 12 |  |
| Hospital stay (days) (Median, IQR) | 12.8  (8.0,17.2) | 15.4  (12.8,24.1) | 0.012 | 13.4  (7.6,18.9) | 21.1  (11.1,23.4) | 0.328 |
| Duration of intensive care unit stay (hours) (Median, IQR) | 10.4  (7.2,63.2) | 24.8  (8.6,196.0) | 0.041 | 8.9  (6.9,50.2) | 27.9  (7.9,395.3) | 0.088 |
| Length of respirator treatment (hours) (Median, IQR) | 5.3  (3.7,25.9) | 13.0  (4.5,118.5) | 0.033 | 5.4  (3.8,28.2) | 13.7  (3.9,174.0) | 0.373 |
| Secondary respiratory failure (%) | 4.6 | 3.2 | 0.745 | 6.9 | 8.3 | 0.872 |
| De novo atrial fibrillation (%) | 2.3 | 3.2 | 0.778 | 0 | 8.3 | 0.116 |
| Thromboembolic event (%) | 4.6 | 6.5 | 0.687 | 0 | 0 | 1 |
| Epilepsy (%) | 1.1 | 0 | 0.549 | 3.4 | 0 | 0.515 |
| Clinical vasospasm (%) | 14.9 | 12.9 | 0.781 | 24.1 | 16.7 | 0.599 |
| Radiological/ultrasonological vasospasm (%)  No vasospasm  Slight to moderate in 1 artery  Slight to moderate in multiple arteries  Severe in 1 artery  Severe multivessel spasm | 52.3  10.5  23.3  7.0  7.0 | 32.3  12.9  41.9  3.2  9.7 | 0.055  0.711  0.048  0.450  0.629 | 41.4  10.3  34.5  3.4  10.3 | 25.0  33.3  16.6  8.3  8.3 | 0.322  0.075  0.254  0.139  0.843 |
| DCI (%) | 6.9 | 9.7 | 0.616 | 13.8 | 16.7 | 0.813 |
| 90-day mortality (%) | 2.3 | **3.2** | 0.758 | 0 | **33.3 p=0.025** | 0.005 |
| Smoking at follow-up (%) | 30.8 | 29.6 | 0.821 | 41.4 | 33.3 | 0.903 |
| Moderate to heavy smokers (>10 cigarettes/day) | | | | | | |
| N= | 70 | 80 |  | 55 | 72 |  |
| Hospital stay (days) (Median, IQR) | 14.9  (10.0,21.6) | 15.3  (11.1,19.9) | 0.837 | 17.4  (9.4,22.9) | 15.9  (11.9,20.8) | 0.996 |
| Duration of intensive care unit stay (hours) (Median, IQR) | 47.7  (8.1,286.4) | 26.3  (10.1,176.8) | 0.371 | 22.8  (7.2,302.4) | 30.4  (8.2,219.4) | 0.717 |
| Length of respirator treatment (hours) (Median, IQR) | 36.5  (4.9,236.5) | 16.9  (4.7,170.7) | 0.362 | 7.6  (4.1,271.1) | 17.2  (5.8,189.5) | 0.426 |
| Secondary respiratory failure (%) | 10.0 | 8.8 | 0.793 | 5.5 | 12.5 | 0.179 |
| De novo atrial fibrillation (%) | 14.3 | 5.0 | 0.051 | 5.5 | 1.4 | 0.194 |
| Thromboembolic event (%) | 8.6 | 6.3 | 0.586 | 5.5 | 11.1 | 0.261 |
| Epilepsy (%) | 0 | 1.3 | 0.348 | 1.8 | 0 | 0.251 |
| Clinical vasospasm (%) | 21.4 | 20.0 | 0.829 | 23.6 | 29.2 | 0.486 |
| Radiological/ultrasonological vasospasm (%)  No vasospasm  Slight to moderate in 1 artery  Slight to moderate in multiple arteries  Severe in 1 artery  Severe multivessel spasm | 35.7  22.9  24.3  5.7  11.4 | 43.8  11.3  26.3  5.0  13.8 | 0.316  0.057  0.783  0.840  0.670 | 43.6  14.5  18.2  10.9  12.7 | 43.1  16.7  23.6  6.9  9.7 | 0.948  0.745  0.459  0.431  0.592 |
| DCI (%) | 11.4 | 10.0 | 0.777 | 23.6 | 18.1 | 0.440 |
| 90-day mortality (%) | 10.4 | 8.1 | 0.632 | 5.9 | 11.9 | 0.262 |
| Smoking at follow-up (%) | 37.3 | 36.1 | 0.740 | 41.8 | 41.7 | 0.912 |

*DCI: delayed cerebral ischemia; NRT: nicotine replacement therapy; VS:vasospasm. In bold: significant difference between females and males with p-value*
